# Supplementary material for: End Sequence Analysis Toolkit (ESAT) expands the extractable information from single-cell RNA-seq data
Source: Genome Res. 2016 Oct;26(10):1397–410. doi: 10.1101/gr.207902.116 (PMC5052061; doi:10.1101/gr.207902.116)
Supplement: Supplemental Material [file supp_gr.207902.116_Supp_Materials.tar.gz › ESAT-esat_v0.1/doc/html/classbroad_1_1core_1_1multiplealignment_1_1_multiple_alignment-members.html]

ESAT: Member List


ESAT


- Main Page
- Related Pages
- Packages
- Classes
- Files

- Class List
- Class Index
- Class Hierarchy
- Class Members

All Classes Namespaces Files Functions Variables Enumerator Pages

- broad
- core
- multiplealignment
- MultipleAlignment

broad.core.multiplealignment.MultipleAlignment Member List

This is the complete list of members for broad.core.multiplealignment.MultipleAlignment, including all inherited members.

|  |  |  |
| --- | --- | --- |
| A\_CODE | broad.core.multiplealignment.MultipleAlignment | static |
| addSequence(AlignedSequence seq) | broad.core.multiplealignment.MultipleAlignment |  |
| addSequences(List< AlignedSequence > sequences) | broad.core.multiplealignment.MultipleAlignment |  |
| addShortEncodedColumn(Map< String, Short > col) | broad.core.multiplealignment.MultipleAlignment |  |
| addShortEncodedColumnMatrix(Map< String, Matrix > col) | broad.core.multiplealignment.MultipleAlignment |  |
| addShortEncodedRegion(Map< String, short[]> region) | broad.core.multiplealignment.MultipleAlignment |  |
| append(MultipleAlignment other) | broad.core.multiplealignment.MultipleAlignment |  |
| C\_CODE | broad.core.multiplealignment.MultipleAlignment | static |
| compress() | broad.core.multiplealignment.MultipleAlignment |  |
| decodeShort(Short nucleotide) | broad.core.multiplealignment.MultipleAlignment |  |
| encode() | broad.core.multiplealignment.MultipleAlignment |  |
| encode(boolean distinguishMissingSequence) | broad.core.multiplealignment.MultipleAlignment |  |
| encodeAsMatrix() | broad.core.multiplealignment.MultipleAlignment |  |
| G\_CODE | broad.core.multiplealignment.MultipleAlignment | static |
| GAP\_CODE | broad.core.multiplealignment.MultipleAlignment | static |
| getAlignedSequence(String containingSequenceId) | broad.core.multiplealignment.MultipleAlignment |  |
| getAlignedSequenceIds() | broad.core.multiplealignment.MultipleAlignment |  |
| getAlignedSequences() | broad.core.multiplealignment.MultipleAlignment |  |
| getAlignedSequences(String refId, int refStart, int refEnd, boolean reverse) | broad.core.multiplealignment.MultipleAlignment | protected |
| getAsMatrix() | broad.core.multiplealignment.MultipleAlignment |  |
| getColumn(int i) | broad.core.multiplealignment.MultipleAlignment |  |
| getColumnsAsVector(int start, int number) | broad.core.multiplealignment.MultipleAlignment |  |
| getConcatenatedSubAlignment(String refId, List<?extends GenomicAnnotation > annotations) | broad.core.multiplealignment.MultipleAlignment |  |
| getIOHelper() | broad.core.multiplealignment.MultipleAlignment | protected |
| getNumberOfAligningSequences() | broad.core.multiplealignment.MultipleAlignment |  |
| getReference() | broad.core.multiplealignment.MultipleAlignment |  |
| getReferenceEnd() | broad.core.multiplealignment.MultipleAlignment |  |
| getReferenceId() | broad.core.multiplealignment.MultipleAlignment |  |
| getReferenceStart() | broad.core.multiplealignment.MultipleAlignment |  |
| getScore() | broad.core.multiplealignment.MultipleAlignment |  |
| getSubAlignment(int refStart, int refEnd, boolean reverse) | broad.core.multiplealignment.MultipleAlignment |  |
| getSubAlignment(String refId, int refStart, int refEnd, boolean reverse) | broad.core.multiplealignment.MultipleAlignment |  |
| getUngappedReferenceIslands() | broad.core.multiplealignment.MultipleAlignment |  |
| getUngappedSequenceReferenceIslands() | broad.core.multiplealignment.MultipleAlignment |  |
| introduceGaps(boolean canGapReference, int maxNumberOfGaps) | broad.core.multiplealignment.MultipleAlignment |  |
| isEmpty() | broad.core.multiplealignment.MultipleAlignment |  |
| isRefGapped() | broad.core.multiplealignment.MultipleAlignment |  |
| length() | broad.core.multiplealignment.MultipleAlignment |  |
| MultipleAlignment() | broad.core.multiplealignment.MultipleAlignment |  |
| MultipleAlignment(List<?extends MultipleAlignment > alnBlocks, String ref, String[] seqs) | broad.core.multiplealignment.MultipleAlignment |  |
| overlaps(SequenceRegion region) | broad.core.multiplealignment.MultipleAlignment |  |
| permuteColumns() | broad.core.multiplealignment.MultipleAlignment |  |
| remove(List< String > removeList) | broad.core.multiplealignment.MultipleAlignment |  |
| reverse() | broad.core.multiplealignment.MultipleAlignment |  |
| sampleColumns(int colNum, int numConsecutiveCols) | broad.core.multiplealignment.MultipleAlignment |  |
| setIOHelper(MultipleAlignmentIO maio) | broad.core.multiplealignment.MultipleAlignment |  |
| setReferenceId(String referenceId) | broad.core.multiplealignment.MultipleAlignment |  |
| setRefGapped(boolean isRefGapped) | broad.core.multiplealignment.MultipleAlignment |  |
| setScore(float score) | broad.core.multiplealignment.MultipleAlignment |  |
| T\_CODE | broad.core.multiplealignment.MultipleAlignment | static |
| UNGAPPED\_ALPHABET\_SIZE | broad.core.multiplealignment.MultipleAlignment | protectedstatic |
| write(BufferedWriter bw) | broad.core.multiplealignment.MultipleAlignment |  |
| write(BufferedWriter bw, List< String > order) | broad.core.multiplealignment.MultipleAlignment |  |


---

Generated on Fri Aug 15 2014 15:10:55 for ESAT by  

 1.8.7
